# A portable articulated dynamometer for ankle dorsiflexion and plantar flexion strength measurement: a design, validation, and user experience study

**DOI:** 10.1038/s41598-023-49263-2

**Published:** 2023-12-14

**Authors:** Seung Yeon Cho, Youho Myong, Sungwoo Park, Minwoo Cho, Sungwan Kim

**Affiliations:** 1 Interdisciplinary Program in Bioengineering, The Graduate School, Seoul National University, Seoul, South Korea; 2 Department of Biomedical Engineering, Seoul National University College of Medicine, 103 Daehak-Ro, Jongno-Gu, Seoul, 03080 South Korea; 3 Department of Rehabilitation Medicine, Seoul National University Hospital, Seoul, South Korea; 4 Institute of Innovative Medical Technology, Seoul National University Hospital Biomedical Research Institute, Seoul, South Korea; 5 Department of Transdisciplinary Medicine, Seoul National University Hospital, 101 Daehak-Ro, Jongno-Gu, Seoul, 03080 South Korea; 6 Department of Medicine, Seoul National University College of Medicine, Seoul, South Korea; 7 Institute of Bioengineering, Seoul National University, Seoul, South Korea

**Keywords:** Biomedical engineering, Diagnosis

## Abstract

Monitoring ankle strength is crucial for assessing daily activities, functional ability, and preventing lower extremity injuries. However, the current methods for measuring ankle strength are often unreliable or not easily portable to be used in clinical settings. Therefore, this study proposes a portable dynamometer with high reliability capable of measuring ankle dorsiflexion and plantar flexion. The proposed portable dynamometer comprised plates made of aluminum alloy 6061 and a miniature tension–compression load cell. A total of 41 healthy adult participants applied maximal isometric dorsiflexor and plantar flexor forces on a Lafayette Handheld Dynamometer (HHD) and the portable dynamometer. The results were cross-validated, using change in mean, and two independent examiners evaluated the inter-rater and test–retest reliabilities in separate sessions using intraclass correlation coefficients, standard error of measurement, and minimal detectable change. Both dorsiflexion and plantar flexion measurements demonstrated a strong correlation with the HHD (r = 0.827; r = 0.973) and showed high inter-rater and test–retest reliabilities. Additionally, the participant responses to the user experience questionnaire survey indicated vastly superior positive experiences with the portable dynamometer. The study findings suggest that the designed portable dynamometer can provide accurate and reliable measurements of ankle strengths, making it a potential alternative to current methods in clinical settings.

## Introduction

Maintaining muscle strength is crucial for overall health and physical fitness, as it plays a vital role in performing daily activities and maintaining functional ability^1–3^. The decline in muscle strength is associated with neuromuscular disorders, and considered a risk factor for all-cause mortality in healthy populations^4–6^. Inadequate muscle strength in the ankle can increase the risk of ankle sprain and other lower extremity injuries since ankle strength is essential for gait and balance^7–9^. For instance, ankle dorsiflexor strength, an important determinant of walking endurance^7^, is often impaired in stroke, cerebral palsy, and other neuromuscular disorders, such as myotonic dystrophy type 1^10^. Moreover, ankle dorsiflexion was found to be one of the primary predictors of loss of ambulation in Duchenne muscular dystrophy^11^, with its weakness also influencing motor function in Charcot-Marie-Tooth disease^12^. Therefore, monitoring the muscle strength of ankles may provide valuable information to clinicians and individuals seeking to improve lower extremity strength and stability, necessitating the appropriate measurement of ankle strength^13^.

Manual muscle testing (MMT) has been the preferred method for ankle-strength measurements in most clinical settings, as it is quick and easy to perform^14,15^. However, its reliability is low because its grading depends on the examiner’s muscle strength, and quantifying temporal changes is challenging^16^. Hand-held dynamometers (HHDs) or the isokinetic dynamometers are commonly used for muscle strength assessment in clinical settings^17–20^. HHD is advantageous over MMT because it is fully quantitative and more sensitive for detecting small changes^21^; however, its reliability is also limited by the examiner’s strength^22^. Furthermore, HHDs require different subject positioning for different measurements^23,24^. While isokinetic dynamometers are often used as the gold standard for muscle strength assessment, they are significantly larger and less portable, making routine clinical practice or bedside monitoring difficult^20^. Additionally, isokinetic dynamometers are typically more expensive, requiring trained technical expertise owing to the complexity of the software and the setup process^18^.

The existing methods of ankle strength assessment have limitations, with previous studies having attempted to develop a dynamometry system specifically for the measurement of ankle dorsiflexion and plantar flexion strengths. However, some portable dynamometers were limited to only ankle plantar flexion measurements, while others that allowed both measurements demonstrated low reliability^25,26^. Another study developed a novel portable iso-damping dynamometer that was comparable to the gold standard but required a subject-specific pre-parameter set for each assessment, and test–retest validation was absent^27^. Therefore, a portable, highly accurate, and reliable dynamometer that can measure ankle dorsiflexion and plantar flexion is required. Recently, the authors have developed a portable dynamometer with high accuracy and reliability for measurement of knee extensor strength^28^, and the concept was further developed to extend its application to ankle in this study.

This study aimed to design and validate a portable and reliable isometric dynamometer for measuring ankle dorsiflexion and plantar flexion strengths. The accessibility, portability, and reliability of the device were evaluated through cross-validation with healthy adult subjects, comparing the results with those obtained using the Lafayette HHD and followed by a user experience questionnaire survey (UEQ-S). To provide stability during isometric measurements, a Lafayette Support Stand was used in conjunction with the Lafayette HHD.

## Results

A total of 41 healthy adults were included in this study, and the cohort size was sufficient for the validation of proposed device, taking account of the population recruited in similar previous works^17,29^. All subjects performed ankle dorsiflexion and plantar flexion assessments for both ankles in two independent sessions, all of which were evaluated by two different examiners. The results of this study provided 656 observations in total: 328 dorsiflexions and 328 plantar flexions.

**Figure 1:**
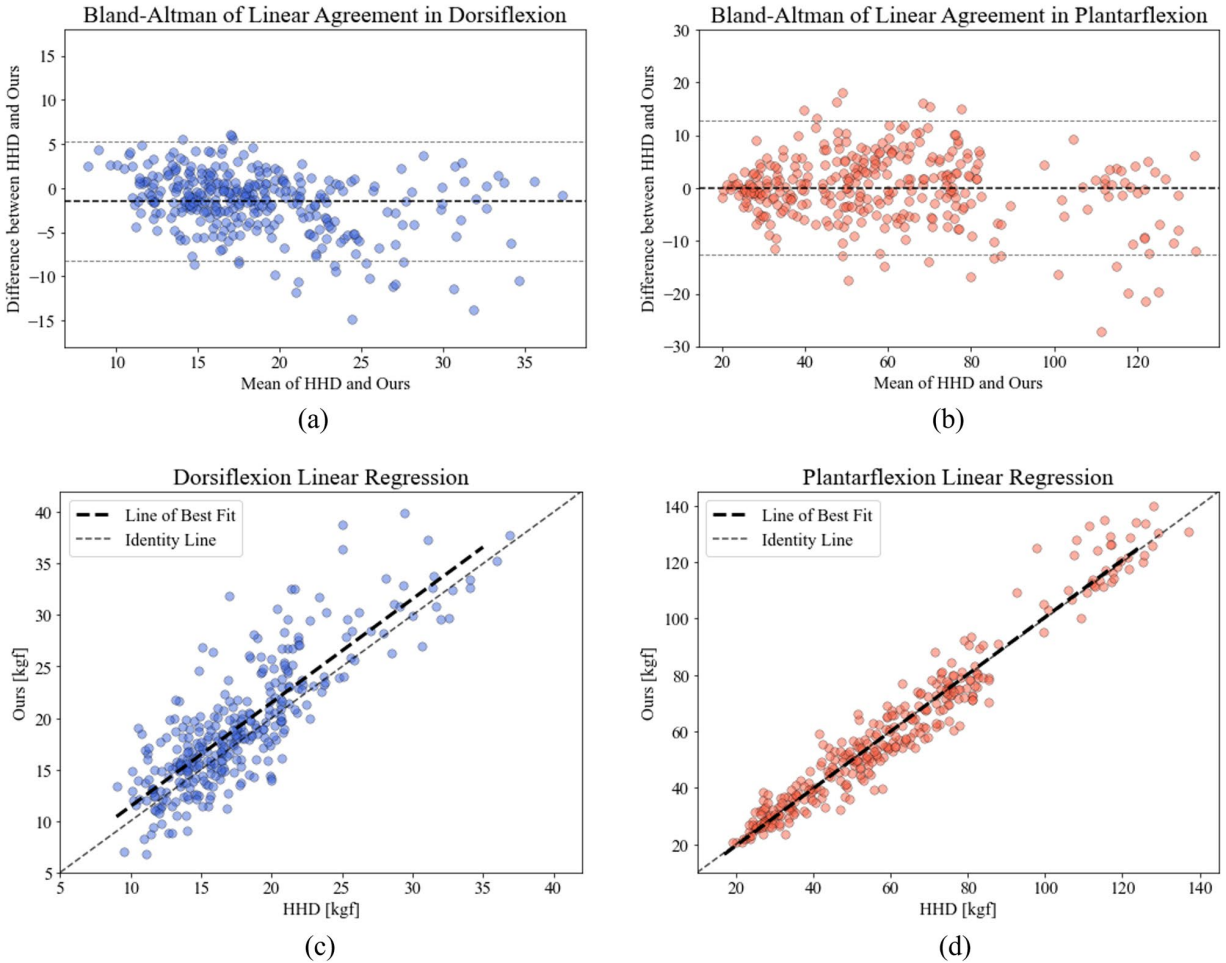
Bland–Altman analyses of the agreement between the portable dynamometer and HHD for (**a**) dorsiflexion and (**b**) plantar flexion measurements; linear regression analyses of the agreement between the portable dynamometer and HHD for (**c**) dorsiflexion and (**d**) plantar flexion measurements.

Table 1 summarizes the results of a cross-validation analysis conducted to assess the agreement between the HHD and the portable dynamometer. The maximum force of ankle dorsiflexion measured with the portable dynamometer exhibited a high correlation (r = 0.827, *p* < 0.001) than that measured with the HHD (mean difference: 1.48 kg f, 95% CI 0.62, and 2.35 kg f). Similarly, the maximum force of ankle plantar flexion measured with the portable dynamometer demonstrated a higher correlation (r = 0.973, *p* < 0.001) than the measurements from the HHD, with no significant difference (mean difference: − 0.04 kg f, 95% CI –4.27, and 4.18 kg f). The intraclass correlation coefficient (ICC) between the HHD and portable dynamometer was 0.785 and 0.972 for the dorsiflexion and plantar flexion, respectively, indicating good and excellent reliability, respectively. The minimal detectable change (MDC) was 7.93 kg f in dorsiflexion and 13.03 kg f in plantar flexion. Figure 1 illustrates the linear relationship between the measured values of the two dynamometers, along with the Bland–Altman analysis^30,31^.

**Table 1:** Summary of overall results: agreement, inter-rater reliability, and test–retest reliability of hand-held dynamometer and portable dynamometer (ours).

	Measurement (kg·f)		CIM (kg·f) (95% CI)	Pearson’s *r*	*p*-value	ICC^a^	SEM (kg·f)	MDC (kg·f)
Agreement								
Dorsiflexion								
HHD	17.69 ± 5.06		1.48 (0.62, 2.35)	0.827	< 0.001	0.785	2.86 (14.92%)	7.93 (41.35%)
Ours	19.17 ± 6.16							
Plantar flexion								
HHD	59.98 ± 27.00		− 0.04 (− 4.27, 4.18)	0.973	< 0.001	0.972	4.70 (7.84%)	13.03 (21.72%)
Ours	59.94 ± 28.11							
Inter-rater reliability								
Dorsiflexion	Examiner A	Examiner B						
HHD	17.61 ± 5.08	17.77 ± 5.06	0.16 (− 0.95, 1.26)	0.941	< 0.001	0.941	1.23 (6.92%)	3.41 (19.19%)
Ours	18.96 ± 6.20	19.39 ± 6.13	0.43 (− 0.90, 1.78)	0.931	< 0.001	0.929	1.63 (8.41%)	4.52 (23.30%)
Plantar flexion								
HHD	59.87 ± 26.65	60.10 ± 27.42	0.23 (− 5.65, 6.10)	0.987	< 0.001	0.986	3.24 (5.39%)	8.98 (14.94%)
Ours	59.71 ± 28.01	60.17 ± 28.29	0.46 (− 5.66, 6.57)	0.969	< 0.001	0.969	4.98 (8.28%)	13.80 (22.94%)
Test–retest reliability								
Dorsiflexion	Session 1	Session 2						
HHD	17.68 ± 5.27	17.70 ± 4.87	0.02 (− 1.09, 1.12)	0.878	< 0.001	0.876	1.71 (9.66%)	4.74 (26.78%)
Ours	19.08 ± 6.49	19.27 ± 5.84	0.19 (− 1.15, 1.53)	0.871	< 0.001	0.867	2.13 (11.05%)	5.90 (30.64%)
Plantar flexion								
HHD	60.35 ± 27.06	59.62 ± 27.02	− 0.73 (− 6.60, 5.14)	0.968	< 0.001	0.968	4.83 (8.00%)	13.39 (22.18%)
Ours	59.73 ± 27.80	60.15 ± 28.50	0.42 (− 5.70, 6.53)	0.961	< 0.001	0.961	5.56 (9.24%)	15.41 (25.62%)

*CIM* change in mean, *ICC* intraclass correlation coefficient, *SEM* standard error of measurement, *MDC* minimal detectable change, *HHD* hand-held dynamometer.

^a^ICC_(2,k)_ was used for the agreement and inter-rater reliability, and ICC_(3,k)_ was used for the test–retest reliability.

Two types of error sources were observed in the result, which can be categorized into objective and subjective error sources. The objective error sources primarily encompass systematic errors, such as calibration errors in the portable dynamometer, as shown in Supplementary Fig. 1. The amount of deviation for a best fit line in the calibration curve indicated the presence of the objective error source. The impact of such objective error source became evident in the linear regression analysis of dorsiflexion measurement (Fig. 1c), wherein a slight force offset was observed.

The subjective error sources would include the variability in measurement setup and data recording performed by examiners, variability in the characteristics of individuals, and potential learning effects over multiple trials. These errors can be analyzed with evaluation of inter-rater reliability and test–retest reliability. The inter-rater reliability of the portable dynamometer was evaluated by having two independent examiners assess each participant. The data collected from both examiners showed a strong correlation with both the portable dynamometer (r = 0.931, *p* < 0.001 in dorsiflexion; r = 0.969, *p* < 0.001 for plantar flexion) and the HHD (r = 0.941, *p* < 0.001 in dorsiflexion; r = 0.987, *p* < 0.001 in plantar flexion). No significant differences between the examiners were observed for either device, with mean differences of 0.16 (dorsiflexion), 0.23 (plantar flexion) and 0.43 (dorsiflexion), 0.46 (plantar flexion) kg f for the HHD and the portable dynamometer, respectively. Both devices demonstrated excellent relative reliability, with ICC values of 0.941 (dorsiflexion), 0.986 (plantar flexion) and 0.929 (dorsiflexion), 0.969 (plantar flexion) for the HHD and the portable dynamometer, respectively. The MDC was 3.41 kg f for the HHD and 4.52 for the portable dynamometer in dorsiflexion, and 8.98 kg f for the HHD and 13.80 kg f for the portable dynamometer in plantar flexion.

**Figure 2:**
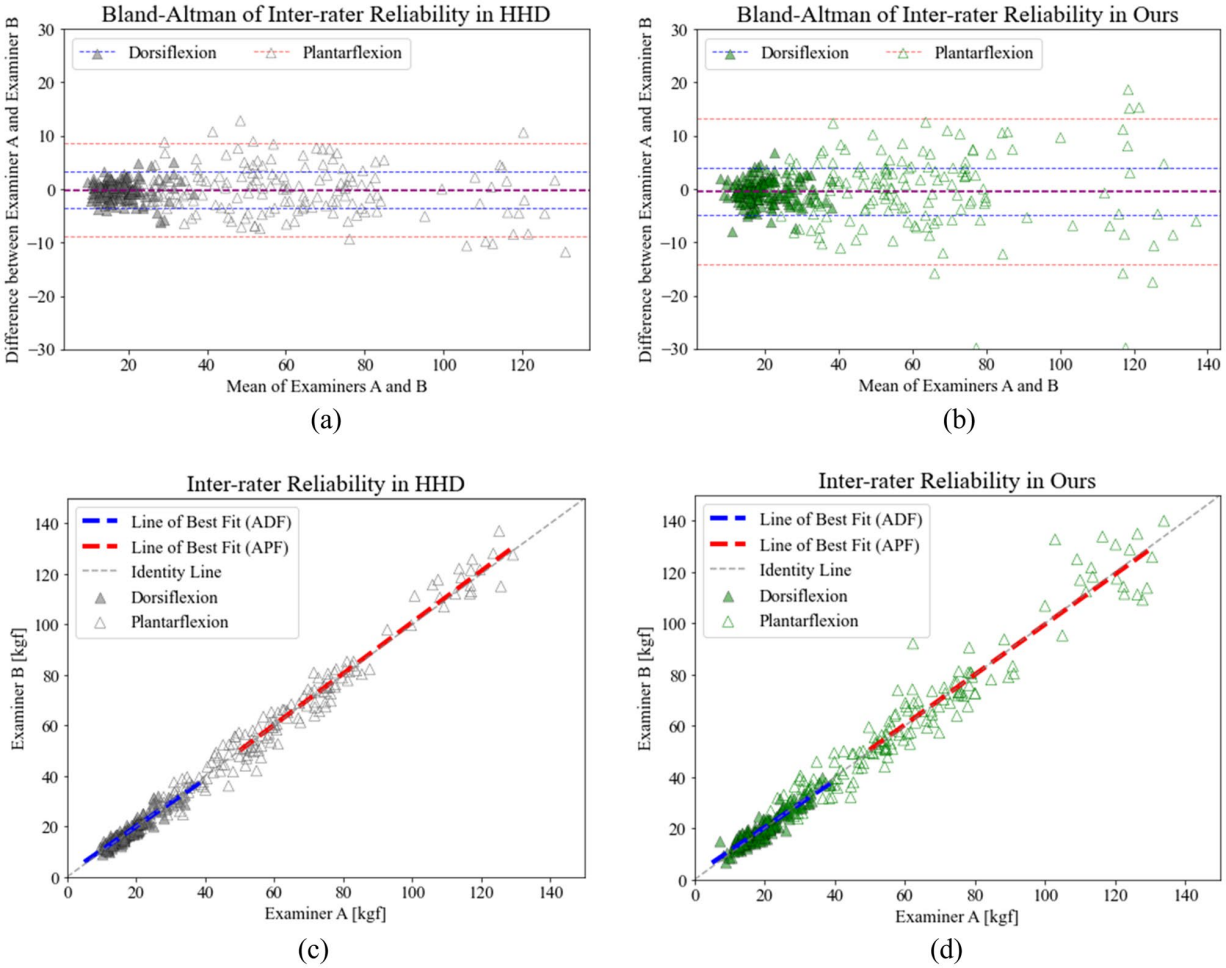
Bland–Altman analyses of the inter-rater reliability for (**a**) HHD and (**b**) portable dynamometer; linear regression analyses of the inter-rater reliability for (**c**) HHD and (**d**) portable dynamometer.

**Figure 3:**
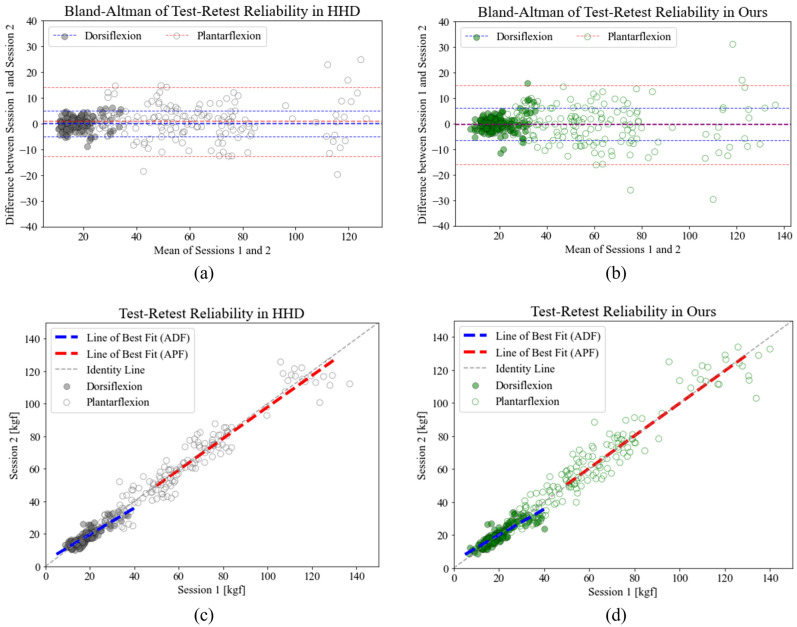
Bland–Altman analyses of the test–retest reliability for (**a**) HHD and (**b**) portable dynamometer; linear regression analyses of the test–retest reliability for (**c**) HHD and (**d**) portable dynamometer.

The study additionally evaluated test–retest reliability by including a retest session after 24 h of the initial session The measurements obtained from the retest session showed a strong correlation with those from the initial session for both the HHD (r = 0.878, *p* < 0.001 in dorsiflexion; r = 0.968, *p* < 0.001 in plantar flexion) and the portable dynamometer (r = 0.871, *p* < 0.001 in dorsiflexion; r = 0.961, *p* < 0.001 in plantar flexion). The between-day measurements for both devices did not have a significant difference, with mean differences of 0.02 (dorsiflexion), − 0.73 (plantar flexion) and 0.19 (dorsiflexion), 0.42 (plantar flexion) kg f for the HHD and portable dynamometer, respectively. Both devices demonstrated excellent relative reliability, with ICC values of 0.876 (dorsiflexion) and 0.968 (plantar flexion) for the HHD, and 0.867 (dorsiflexion) and 0.961 (plantar flexion) for the portable dynamometer. The MDC was 4.74 kg f for the HHD and 5.90 kg f for the portable dynamometer in dorsiflexion, and 13.39 kg f for the HHD and 15.41 for the portable dynamometer in plantar flexion. Table 1 summarizes the results of the inter-rater and test–retest reliabilities, while Figs. 2 and 3 provide a visual representation of the results.

Out of 41 participants in this study, 38 completed the UEQ-S survey. The results reported that the overall user experience with the portable dynamometer was significantly more positive than with the HHD (*p* < 0.001). Specifically, five items showed statistically significant differences between the two devices. Participants found the portable dynamometer to be easier to handle (*p* = 0.004), more efficient (*p* < 0.001), clearer (*p* = 0.032), more inventive (*p* < 0.001), and more leading-edge (*p* = 0.004) than the HHD. Table 2 presents additional details on the survey.

**Table 2:** User experience questionnaire survey score (N = 38).

	HHD	Ours^a^	CIM [95% CI]	*p*-value
Supportive	1.08 ± 1.57	1.66 ± 1.19	0.58 [− 0.06, 1.21]	0.064
Easy	**0.79 ± 1.95**	**2.00 ± 1.34**	**1.21 [0.45, 1.97]**	**0.004**
Efficient	**0.39 ± 1.95**	**1.84 ± 1.13**	**1.45 [0.72, 2.18]**	**< 0.001**
Clear	**0.84 ± 1.84**	**1.63 ± 1.51**	**0.79 [0.02, 1.56]**	**0.032**
Exciting	0.79 ± 1.40	1.05 ± 1.41	0.26 [− 0.38, 0.91]	0.287
Interesting	0.95 ± 1.43	1.18 ± 1.43	0.23 [− 0.42, 0.89]	0.152
Inventive	**0.05 ± 1.77**	**1.55 ± 1.41**	**1.50 [0.77, 2.23]**	**< 0.001**
Leading edge	**0.00 ± 1.76**	**0.92 ± 1.38**	**0.92 [0.20, 1.64]**	**0.004**
Total score	**4.89 ± 0.41**	**11.83 ± 0.39**	**6.94 [6.55, 7.33]**	**< 0.001**

*HHD* hand-held dynamometer, *CIM* change in mean, *CI* confidence interval.

Significant values are in bold.

^a^Ours: the portable dynamometer.

## Discussion

The measurement of maximal ankle dorsiflexion and plantar flexion strength is crucial for assessing gait, balance, and other daily activities^1,7,8^. To address the need for accurate and accessible measurements, a portable dynamometer was developed in this study, which aimed to provide portability while improving accessibility and reliability. A sample of 41 healthy adult subjects was considered to test the dynamometer, and its validity was evaluated by assessing the level of agreement with the gold standard, inter-rater reliability, test–retest reliability, and user experience. Isometric measurements were conducted using the HHD, which was fastened to the Lafayette Support Stand to improve stability and objectivity. The HHD showed high reliability in the inter-rater and test–retest assessments, confirming its validity. Although valid, since HHD requires different arrangement or position of either the device or the subject for measurement of different force types, the position of HHD had to be rearranged for each assessment of dorsiflexion and plantar flexion. However, the size (610 × 610 × 880 mm in dimension) and weight (approximately 20 kg) of the stand made rearrangements transportation challenging. Additionally, some subjects experienced discomfort with use of HHD because the point of contact with the device was minimal.

Several studies have previously attempted to increase the portability and reliability of existing ankle muscle strength assessment methods. For instance, a study designed a unique dynamometer that could measure both ankle dorsiflexion and plantar flexion, offered easy stabilization of the ankle joint for strength measurement, and exhibited low variability in healthy subjects^32^. However, the device lacked portability, with its maximal range of measurement too low to cover the plantar flexor force, which is usually larger than that of the subjects they evaluated^33^. Another study developed a custom-built electronic dynamometer for ankle joint torque measurement that used a simple load cell to assess both dorsiflexion and plantar flexion. However, it also lacked portability and had stability problems, where the optimal pivotal position must be determined for every subject, and its validation was conducted using only 4 human subjects^34^. The portable dynamometer developed in this study utilizes a low-cost high-capacity load cell that can sufficiently measure the plantar flexor force of a healthy adult. Additionally, by using inelastic Velcro belts, the ankle joint can be easily stabilized on the device, contributing to the precision of measurements. While another study aimed to develop a portable dynamometry system and validate it on a wider range of subjects from 5 to 80 years of age, the possible positioning of the subject was limited to only one posture, and the test–retest agreement and reliability found in the current study were significantly higher^26^. It is also noteworthy that some studies have measured force of ankles^24,35^, while others have focused on torque measurements^26,34^. Despite such difference, they were all applied for similar diagnostic purposes. This suggests that in clinical settings, force and torque are sometimes interchangeable. However, from an engineering perspective, it is important to consider that torque sensors are typically more expensive, bulkier, and heavier compared to load cells. Given the primary objective of this study, which was to design a portable dynamometer, the proposed device employed a load cell to measure ankle force, allowing for lightweight and portability.

The portable dynamometer used in this study was designed to address the limitations of previous studies. It has succeeded in demonstrating strong agreement with the clinical gold standard HHD, excellent inter-rater reliability, and excellent test–retest reliability for maximal strength measurement of ankle dorsiflexion and plantar flexion. Furthermore, the participants reported significantly more positive experiences with the portable dynamometer in the user experience survey. Compared to an HHD, the portable dynamometer allowed for both ankle dorsiflexion and plantar flexion assessments to be performed in a single position once it was attached to the ankle, eliminating the need for separate positions and arrangements. Moreover, the device was designed to be intuitive and compact, measuring 256 × 94 × 85 mm in dimension when completely folded and weighing only 2.3 kg. This weight is significantly lighter than other portable dynamometer designs previously attempted, which ranged from 3 to 30 kg^27,32^. The use of aluminum material contributed to a lighter weight while maintaining rigidity.

The designed portable dynamometer demonstrated excellent accuracy and reliability in ankle strength measurements in both dorsiflexion and plantar flexion, despite ankle plantar flexion being generally more difficult to measure due to the extent of its strength^33^. However, the agreement for dorsiflexion was relatively lower, which may be due to discomfort in the positioning of the subject, reflecting potential subjective error sources. Ankle dorsiflexion is known to be maximal when the ankle is plantarflexed by 10°–15°^36,37^. However, the portable dynamometer stabilizes the ankle in a neutral position (0°), which may not be the most comfortable position for some subjects, while the HHD may have allowed the subject to assume the most comfortable position because it did not completely secure the ankle in a fixed position. Despite the stretching sessions being preceded by the assessments, some subjects expressed hamstring strains during the dorsiflexion measurement in a long-sitting position, with the ankle in a neutral position, which may have hindered their maximal force production. In addition, a few subjects with larger foot sizes expressed discomfort with the location of the Velcro strap that runs over the dorsum of the foot. Interestingly, some of the dorsiflexion data with a lower agreement with HHD were observed in male subjects with large foot sizes.

In future studies, it is important to address the limitations of this study. One limitation is the customization of the footplate and location of the strap over the dorsum of the foot. Few subjects with relatively larger foot sizes have expressed discomfort with the location of the strap, but the location of strap was able to be adjusted within only a limited range due to given size of the footplate. The strap was limited for large range of adjustment also to strongly fasten the foot, but since the strap has provided sufficient fastening, it could be considered for allowing more adjustment afterwards. This can be improved by customizing the height of the footplate to support larger foot sizes and widening the range of the strap's location to cover the optimal dorsal region of the foot based on its size. Another limitation is the need for additional validation regarding other postures to explore the optimized positions of the ankle and knee for dorsiflexion and plantar flexion measurements. Given that the portable dynamometer allows measurements in seated or supine positions as well, it would be valuable to consider the possible variance in ankle strength measurements in other postures. Potential difference in force characteristics could be observed in other postures for different individuals. Thus, comparisons with HHD in other positions will further enhance the validity of this work. This study only recruited healthy adults aged between 20 and 39 years, so further validation with a more diverse population, including children, elderly individuals, and patients with weak ankle strength, can improve the device's accuracy and reliability.

## Conclusion

The portable dynamometer developed in this study addresses the clinical need for a reliable and accessible device for accurate ankle strength measurements. With its strong agreement with the gold standard HHD and high inter-rater and test–retest reliabilities, this device may be a valuable tool for routine muscle strength monitoring in clinical settings. It has the potential to provide accurate and reliable measurements of muscle strength and can be used in various populations, including children, elderly individuals, and patients with weak ankle strength, after further validation.

## Methods

### Study subjects

**Figure 4:**
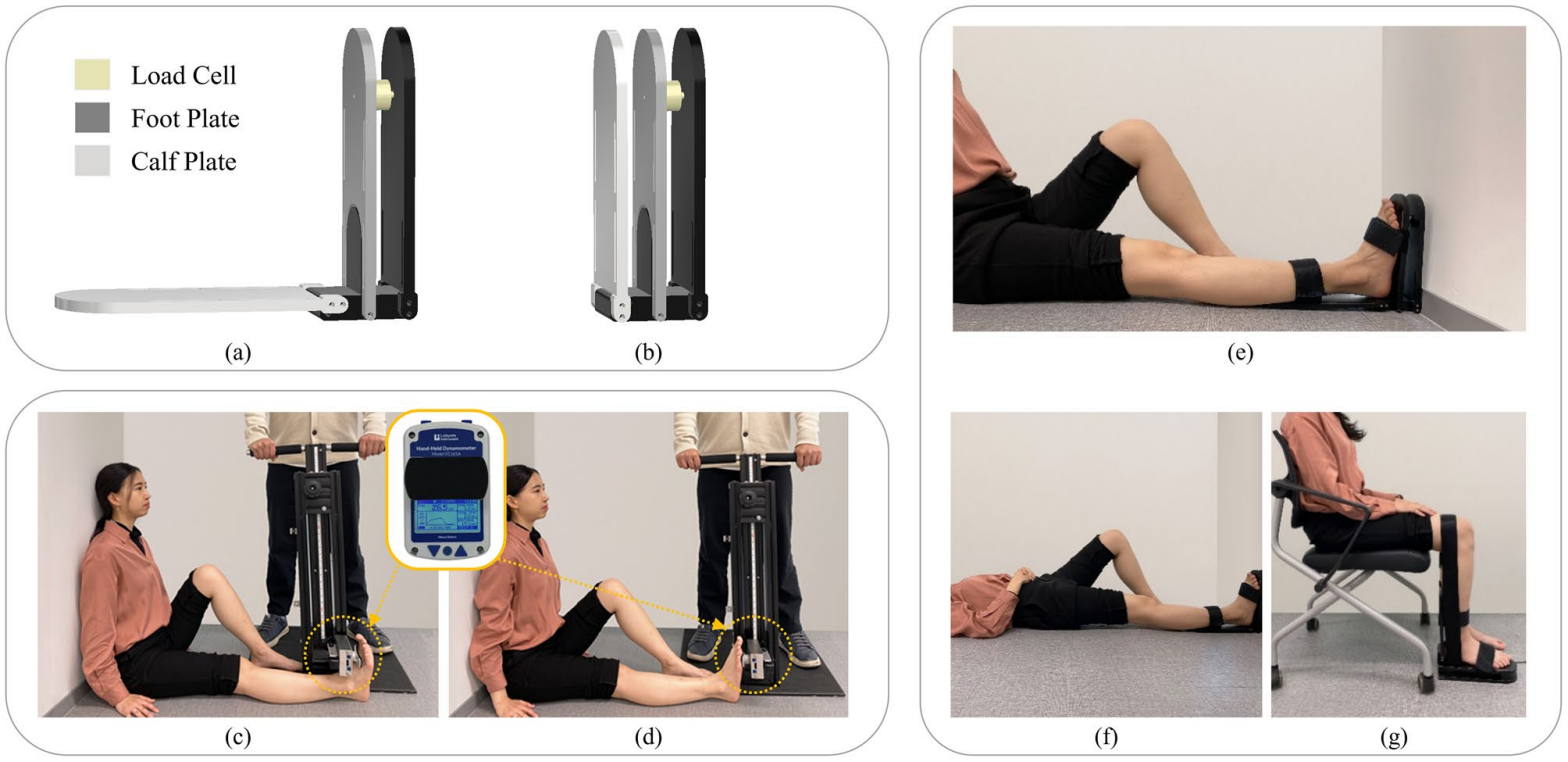
(**a**) 90° formation and (**b**) folded formations of the designed portable dynamometer; subject position for (**c**) ankle dorsiflexion strength measurement and (**d**) ankle plantar flexion strength measurement using the HHD; (**e**) long sitting, (**f**) supine, and (**g**) seated positions for ankle strength measurement using a portable dynamometer. (Figure of HHD is presented within (**c**) and (**d**)).

This study recruited 41 healthy adults (21 males and 20 females) aged between 20 and 39 years who had provided written informed consent (Table 3). Individuals who had undergone lower extremity joint surgery, had neurological, musculoskeletal, or orthopedic conditions, or were unable to understand the instructions were excluded. The study adhered to the principles of the Declaration of Helsinki, with the study protocols approved by the Institutional Review Board Committee of Seoul National University Hospital (IRB number: H-2201-025-1288), Seoul, Korea. The participant appearing in Fig. 4 provided written informed consent to publish the figure in an online open-access publication.

**Table 3:** Demographics and anthropometry of study participants (N = 41).

	Male (N = 21)	Female (N = 20)
Age group (years)		
20–29	20 (49%)	
30–39	21 (51%)	
Age (years)	28.81 ± 4.35	29.95 ± 3.90
Height (cm)	174.31 ± 4.64	162.28 ± 6.34
Weight (kg)	73.86 ± 10.01	53.71 ± 8.93
Foot size (mm)		
Right	249.57 ± 10.22	228.7 ± 9.69
Left	250.33 ± 9.21	228.1 ± 9.75
Grip strength (kg)		
Right	44.29 ± 7.18	27.46 ± 5.00
Left	42.68 ± 7.43	25.95 ± 4.80

*cm* centimeter, *kg* kilogram, *mm* millimeter.

### Anthropometry

The anthropometric data, including height, weight, and foot size, along with birth date and sex, were recorded for each participant (Table 3). This allowed the examiners to identify individuals, while all data were anonymized during the analyses. Height measurements were taken using an ultrasonic stadiometer to the nearest 0.1 cm, with weight measured using a digital scale to the nearest 0.1 kg. Foot sizes were measured (with the subject barefoot) as the distance from the outermost part of the heel to the tip of the longest toe, using a plastic tape measure. Additionally, the maximum grip strength was measured to the nearest 0.1 kg for each hand using a hand-held grip dynamometer (EH-101; Camry Scale, California, USA). The participants were instructed to squeeze the device as hard as possible in an upright posture for approximately 3 s. The foot size and the maximum grip strength were measured for each foot and hand.

### Design and development

The purpose of this study was to design and validate a portable isometric dynamometer that specifically measures ankle dorsiflexion and plantar flexion. The device possessed a simple mechanical structure comprising three main plates made of anodized aluminum alloy 6061, which were joined at a single frame box. A couple of stainless cylinders were included to allow for the device to be folded into a completely compact formation or configured into a 90° angle, with the distal and proximal limbs serving as a footplate and a calf support, respectively. This foldable structure allows for easier storage and enhanced portability. Additionally, a silicone pad was attached at both ends of the calf support plate to maximize wearability and protect the heel and calf from friction during the measurement process, which could potentially cause pain and hinder the subject from applying maximum strength.

**Figure 5:**
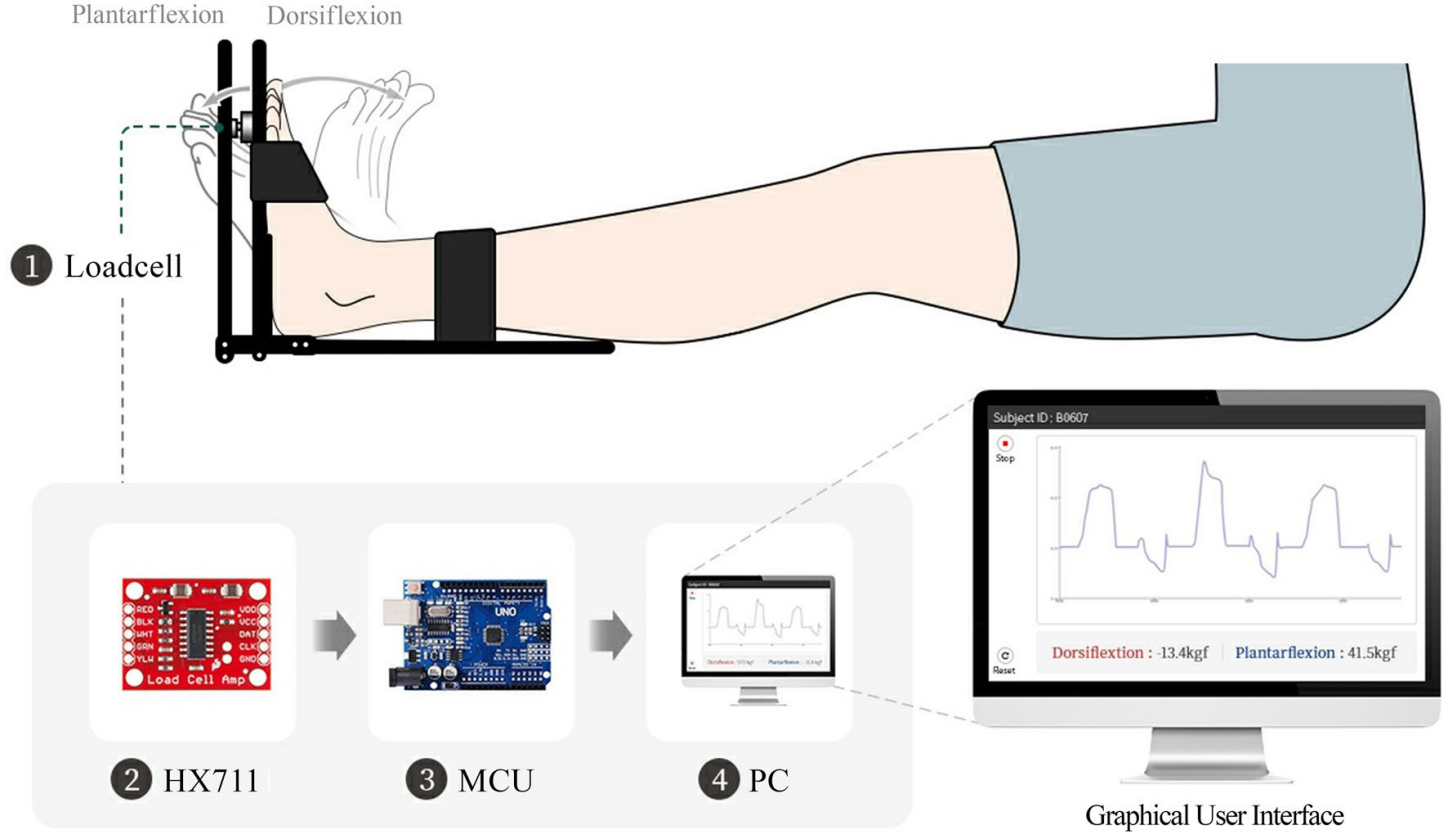
Graphical diagram of the overall flow of the dynamometry system.

A miniature tension–compression load cell (CBF30-100, CASKOREA) was integrated between the two distal limb plates at a distance corresponding to the location of the first metatarsophalangeal joint, as determined by the average foot size of young adults^38^. The signal was acquired at sampling frequency of 10 Hz using an HX711 amplifier and an Arduino Uno microcontroller with analog-to-digital conversion resolution of 10-bit, which amplified and converted the load cell output into a digital signal that could be processed by the microcontroller unit. The acquired data were then transferred to a computer via a serial port connection through an integrated development environment (Arduino IDE 1.8.19) with a baud rate of 9600 bits per second and visualized through a graphical user interface created by Processing 4.0b2. A real-time signal graph was presented for visual assistance in monitoring the signal acquisition, and the maximum value of the acquired data was selected for the final data to record. The overall system had approximately 97.7 g of resolution, and the system flow is illustrated in Fig. 5.

Prior to conducting the clinical trials in human subjects, the load cell was calibrated for force measurements. The calibration was performed by applying a series of M1 class test weights ranging from 0.1 to 20 kg to the dynamometer at the location of the load cell and recording the corresponding readings of the measured value. The applied forces were then correlated with the experimentally measured forces from the device to validate the calibration of the load-cell-embedded device. The results demonstrated strong agreement between the applied and measured forces (r = 0.999, *p* < 0.001), indicating accurate force measurements. The calibration curve is shown in Supplementary Fig. 1.

### Maximal ankle Dorsi-/plantar flexion measurement

To secure the device when the subject exerted force, the HHD (Lafayette Instrument Company, USA, Model 01163) was attached to the Hand-Held Dynamometer Support Stand (Lafayette Instrument Company, USA, Model 01166), hereinafter referred to as the stand, allowing more objective and reliable measurement of the isometric force. The participants were seated on a non-slip floor in a long-sitting position with their back against the wall. The stand was configured to position the HHD in front of the dorsal region of the foot for dorsiflexion measurement, and behind the plantar region of the foot for plantar flexion measurement (Fig. 4c,d). The height of the HHD was adjusted for each participant to align the stirrup with their first metatarsophalangeal joint. The examiner stood on the footplate of the stand and held onto the handlebars to provide additional stability during the measurement. For measurements using the portable dynamometer, a subject's foot was fastened to the device with Velcro belts, and they sat in a long-sitting position with their feet against the wall (Fig. 4e). Although this study conducted the assessments in a long-sitting position, the design of the portable dynamometer allowed measurements in a supine or seated position, as shown in Fig. 4f,g. In the case of measurements in a seated position, the zeroing function of the system, which resets the measurement to zero once the subject is positioned, can eliminate the effect of foot weight. Such feature ensured that measurements can be taken with minimal interference from the external loads in any measurement position.

For both dynamometers, the subjects were instructed to perform maximal dorsiflexion and plantar flexion for a duration of 3 s per force direction. Verbal guidance was provided for the counts, and subjects were encouraged to exert maximum force in each trial. The inter-rater reliability was assessed by two independent examiners (who measured both ankles), and test–retest reliability was evaluated by repeating identical protocols for two sessions, 24 h apart. To prevent muscle fatigue, subjects were given a break of 10 min between the trials of each dynamometer, and a break of 3 min when switching examiners. Additionally, all subjects underwent a stretching session of 3 min to relax their muscles prior to the assessments. The assessments were supervised by a physiatrist with seven years of clinical experience. The overall protocol is illustrated in Supplementary Fig. 2.

### User experience study

After the assessments, the participants were asked to complete the short version of the UEQ-S voluntarily to evaluate their experience with both dynamometers. The questionnaire is a reliable tool for measuring subjective impressions of the user experience with a product^39^. The UEQ-S comprised eight items, each rated on a 7-point Likert scale ranging from − 3 (most negative experience) to + 3 (most positive experience); all responses were anonymous.

### Statistical analysis

The purpose of this study was to evaluate the performance of the designed portable dynamometer in comparison to the HHD, as well as to assess inter-rater reliability and test–retest reliabilities. Statistical analyses were conducted using Pearson’s correlation coefficient, intraclass correlation coefficient (ICC), and the standard error of measurement (SEM), where ICC and SEM represented relative and absolute reliabilities, respectively^40^. ICC values of less than 0.5, between 0.5 and 0.7, between 0.75 and 0.9, and greater than 0.9 represented poor, moderate, good, and excellent relative reliability, respectively^41^. A two-way random-effects model (ICC_2,k_) was used to evaluate the agreement between the two dynamometers and inter-rater reliability, and a two-way mixed effects model (ICC_3,k_) was used to assess the test–retest reliability. The SEM was calculated using the standard deviation (SD), where $SEM = SD \sqrt{1-ICC}$, and minimal detectable change at 95% confidence level was computed using the formula $MDC=1.96\cdot \sqrt{2}\cdot SEM$. Additionally, changes in the mean (CIM) with 95% confidence intervals and paired *t*-tests were used to examine systematic bias. The UEQ-S responses between the HHD and the designed dynamometer were compared using a paired *t*-test. The normal distribution of all data was assessed using the Shapiro–Wilk test. All statistical analyses were performed using the Python package SciPy (v.1.10.1) and its subpackage designed for statistical analyses, scipy.stats^42^. The level of statistical significance was set at *p* < 0.05.

### Supplementary Information

Supplementary Figures.

## Data Availability

The data acquired in this study are not openly available due to the sensitive nature of human data (e.g. age, sex, height, and weight). A de-identified dataset containing the full demographic and clinimetric data is available from the corresponding authors (M.C., S.K.) upon reasonable request.
