# Supplementary material for: A portable articulated dynamometer for ankle dorsiflexion and plantar flexion strength measurement: a design, validation, and user experience study
Source: Sci Rep. 2023 Dec 14;13:22221. doi: 10.1038/s41598-023-49263-2 (PMC10721896; doi:10.1038/s41598-023-49263-2)
Supplement: Supplementary file 1 — Supplementary Figures. [file 41598_2023_49263_MOESM1_ESM.pdf]

# A Portable Articulated Dynamometer for Ankle Dorsiflexion and Plantar Flexion Strength Measurement: A Design, Validation, and User Experience Study

Seung Yeon Cho[1†], Youho Myong[2,3†], Sungwoo Park[1,4], Minwoo Cho[5,6*], Sungwan Kim[1,2,7*]

[1]*Interdisciplinary Program in Bioengineering, The Graduate School, Seoul National University, Seoul, Korea*

[2]*Department of Biomedical Engineering, Seoul National University College of Medicine, Seoul, Korea*

[3]*Department of Rehabilitation Medicine, Seoul National University Hospital, Seoul, Korea*

[4]*Institute of Innovative Medical Technology, Seoul National University Hospital Biomedical Research Institute, Seoul, Korea*

[5]*Department of Transdisciplinary Medicine, Seoul National University Hospital, Seoul, Korea*

[6]*Department of Medicine, Seoul National University College of Medicine, Seoul, Korea*

[7]*Institute of Bioengineering, Seoul National University, Seoul, Korea*

[*]**Correspondence: Sungwan Kim**

Department of Biomedical Engineering, Seoul National University College of Medicine, 103 Daehak-ro, Jongno-gu, Seoul 03080, Korea

E-mail: sungwan@snu.ac.kr

[*]**Co-correspondence: Minwoo Cho**

Department of Transdisciplinary Medicine, Seoul National University Hospital, 101 Daehak-ro, Jongno-gu, Seoul 03080, Korea

E-mail: windsblues@snu.ac.kr

[†] These authors contributed equally to this work.

[*] These authors contributed equally to this work.

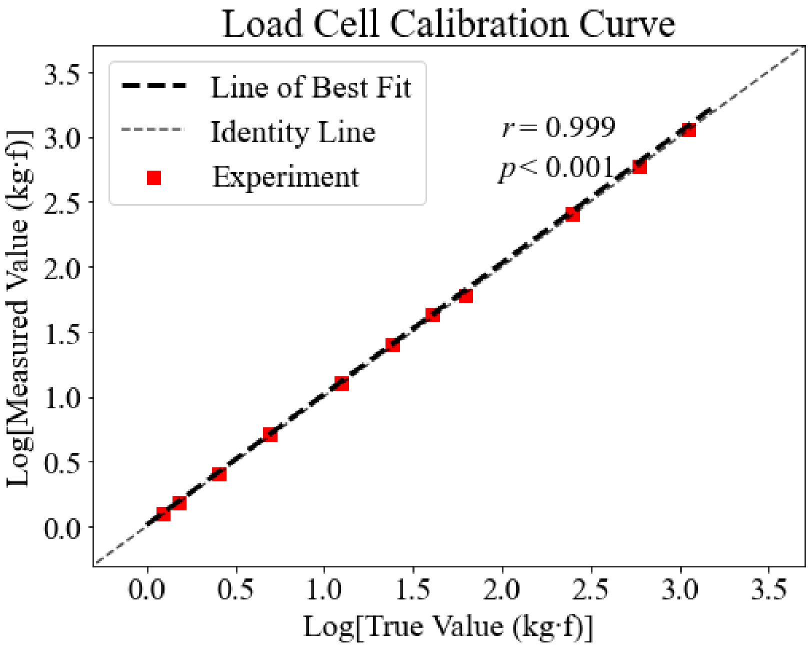


**Supplementary Fig S1.** Calibration curve of the load cell in portable dynamometer.

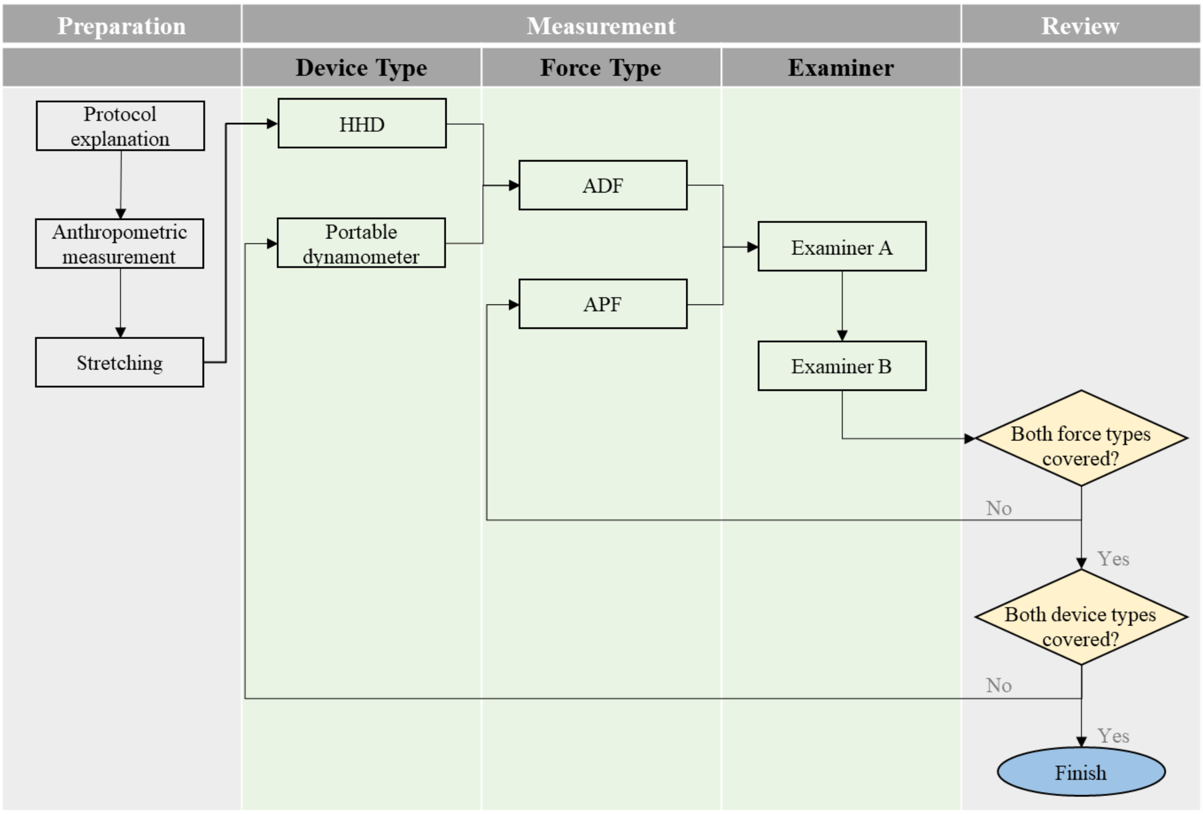


**Supplementary Fig S2.** Flowchart representation of the overall protocols for ankle strength assessment using hand-held dynamometer and portable dynamometer. (HHD: hand-held dynamometer, ADF: ankle dorsiflexion, APF: ankle plantar flexion)
